# Mindfulness-induced selflessness: a MEG neurophenomenological study

**DOI:** 10.3389/fnhum.2013.00582

**Published:** 2013-09-24

**Authors:** Yair Dor-Ziderman, Aviva Berkovich-Ohana, Joseph Glicksohn, Abraham Goldstein

**Affiliations:** ^1^The Leslie and Susan Gonda (Goldschmied) Multidisciplinary Brain Research Center, Bar-Ilan UniversityRamat Gan, Israel; ^2^Department of Neurobiology, Weizmann Institute of ScienceRehovot, Israel; ^3^Department of Criminology, Bar-Ilan UniversityRamat Gan, Israel; ^4^Department of Psychology, Bar-Ilan UniversityRamat Gan, Israel

**Keywords:** self-awareness, minimal self, narrative self, MEG, mindfulness meditation, neurophenomenology, beta frequency band, right inferior parietal lobule

## Abstract

Contemporary philosophical and neurocognitive studies of the self have dissociated two distinct types of self-awareness: a “narrative” self-awareness (NS) weaving together episodic memory, future planning and self-evaluation into a coherent self-narrative and identity, and a “minimal” self-awareness (MS) focused on present momentary experience and closely tied to the sense of agency and ownership. Long-term Buddhist meditation practice aims at realization of a “selfless” mode of awareness (SL), where identification with a static sense of self is replaced by identification with the phenomenon of experiencing itself. NS-mediating mechanisms have been explored by neuroimaging, mainly fMRI, implicating prefrontal midline structures, but MS processes are not well characterized and SL even less so. To this end we tested 12 long-term mindfulness meditators using a neurophenomenological study design, incorporating both magnetoencephalogram (MEG) recordings and first person descriptions. We found that (1) NS attenuation involves extensive frontal, and medial prefrontal gamma band (60–80 Hz) power decreases, consistent with fMRI and intracranial EEG findings; (2) MS attenuation is related to beta-band (13–25 Hz) power decreases in a network that includes ventral medial prefrontal, medial posterior and lateral parietal regions; and (3) the experience of selflessness is linked to attenuation of beta-band activity in the right inferior parietal lobule. These results highlight the role of dissociable frequency-dependent networks in supporting different modes of self-processing, and the utility of combining phenomenology, mindfulness training and electrophysiological neuroimaging for characterizing self-awareness.

## Introduction

An unremitting companion of human experience is the sense of self. Amidst the ocean of coming-and-going waves of perceptions, cognitions and emotions, an absolute certainty regarding the identity of the present-moment experiencer—“self as I”—remains unwavering (James, 1890). On the other hand, the thread of a constant, static, unchanging self—the “self as Me”—stretches back to childhood years, and extends as far into the future as one can imagine. The protagonist of both scenarios is experienced as one-and-the-same, even though the respective (imagined/remembered) bodies, mental capacities, as well as external contexts have completely changed. These phenomenally distinct aspects of self-awareness are being re-conceptualized by contemporary philosophers, psychologists and neurobiologists, aiming at a fruitful exchange between philosophy of mind, phenomenology, and the cognitive sciences. One such influential conceptualization has been offered by Gallagher (2000) as “minimal” and “narrative” forms of self-awareness.

The “minimal” self (MS) is defined as a consciousness of oneself as the immediate subject of experience. It is pre-reflective, present-centered, experiential in nature, and importantly, involves a sense of “ownership” and “agency”: the sense that it is I who is undergoing an experience (Gallagher, 2000). MS, or “core self” in Damasio's (1999, 2010) terms, is understood to be intermittent. Damasio describes it as “… a transient entity, ceaselessly recreated for each and every object with which the brain interacts” (Damasio, 1999, p. 17), in this way implementing a self/non-self distinction and thus specifying the self in perception, cognition, emotion, and action (Christoff et al., 2011). The “narrative” self (NS), on the other hand, refers to a self extended in time, heavily reliant on language, episodic/autobiographical memory and imagination (planned/expected future), and corresponding to identity and personhood. The notion of NS has appeared in the literature under other names such as the “extended” self and “conceptual” self (Neisser, 1988), the “autonoetic” self (Gardiner, 2001) and the “autobiographical” self (Damasio, 1999, 2010), and has been shown to be closely tied to a neurophysiological baseline (Gusnard et al., 2001; Buckner et al., 2008), the so-called default-mode network (DMN, Gusnard and Raichle, 2001; Raichle et al., 2001) and to mind-wandering (Mason et al., 2007; Christoff et al., 2009; Hasenkamp et al., 2012). It is important to note that NS and MS are processes which may operate concurrently. Like other conscious mental content produced by the brain, NS representations, perceived as thoughts and feelings, are stamped with the subjective signature of being *our* thoughts and feelings. Thus, self-specifying processes are at play also during NS (Gallagher, 2000; Damasio, 2010).

Eastern philosophy, and in particular Buddhist philosophy, exhibits a radically different view of the self and personal identity, advocating a “selfless” mode of processing phenomena (SL). At the core of Buddhist psychology lies the teaching of there being no such thing as a permanent, unchanging self (Dreyfus and Thompson, 2007; Olendzki, 2010). The self is understood to be illusory in the sense of being no more than a mental process—and identification with it is understood to be at the very root of suffering. Thus, a primary target of Buddhist practice is the realization of the illusory nature of the self and cultivation of a selfless, boundless mode of experience where identification with a static sense of self is replaced by identification with the phenomenon of experiencing itself (Hart, 1987; Dalai Lama, 1991; Austin, 2000; Ekman et al., 2005; Wallace, 2006; Nydahl, 2008). The notion of relinquishing the sense of owning and directing experience, may seem to the Western mind as nothing short of pathological (for a related discussion, see Engler, 2003). And indeed, pathological brains such as of schizophrenic patients experiencing “thought insertion” (Frith, 1992; Gallagher, 2004) or patients who have suffered lesions (Damasio et al., 2012; Philippi et al., 2012) compromising specific self functions, have largely contributed to Damasio's and Gallagher's understanding of the minimal/core self-concept. In a similar vein, as has been previously suggested (Lutz et al., 2007; Tagini and Raffone, 2010), long-term mindfulness meditators can provide parallel information regarding the self through diminishing the agentive/ownership aspects of present-moment experience. Such information, however, has the advantage of being volitionally produced and in non-diseased brains.

The neurophysiology of NS is relatively well established. A wealth of recent large-scale meta-analyses of mainly fMRI studies investigating self-referential processing through a variety of paradigms have consistently shown it to be modulated by a subset of the DMN, namely the central midline structures, and in particular the medial prefrontal cortex (mPFC) (Northoff et al., 2006, 2011; Buckner et al., 2008; Andrews-Hanna et al., 2010; Qin and Northoff, 2011; Whitfield-Gabrieli et al., 2011; Kim, 2012). Translating these findings into electrophysiological terms, there is accumulating evidence that the fMRI's hemodynamic response signal attributed to DMN and self-referential processing is correlated with neuronal activity in the gamma band (EEG studies—Mantini et al., 2007; Berkovich-Ohana et al., 2012), and in particular high-gamma band (intracranial EEG studies—Nir et al., 2007; Jerbi et al., 2010; Ossandón et al., 2011; Ramot et al., 2012). It should be noted that whether self-referential paradigms can reveal neural activity specific to the self is a matter of current debate, as these tasks involve, and are thus confounded by, higher-order cognitive functions such as evaluation, judgment and reflective thought (Legrand and Ruby, 2009; Christoff et al., 2011; Northoff et al., 2011).

The neural correlates of MS are less well established, with approaches aiming at identifying self-specifying pre/non–reflective processes including merely perceiving, without judgment or evaluation, self-specific vs. non-specific stimuli (Schneider et al., 2008; Northoff et al., 2009), or improving time resolution using event-related EEG (Esslen et al., 2008) or MEG (Walla et al., 2007). Other approaches are informed by phenomenology (Gallagher and Sørensen, 2006) and target MS via one of its core attributes—the sense of agency and ownership (for reviews see David et al., 2008; Sperduti et al., 2011). Key regions here include the inferior parietal lobule (IPL) and the insula. Importantly, the literature does not supply information regarding the oscillatory signature of these mostly fMRI findings. Oscillatory power increases/decreases that occur in specific frequency bands and within different cortical areas have been shown to be functionally relevant in the brain (Singh, 2012). In particular, the different modes of self-awareness might not only involve different brain topographies, but perhaps also different oscillatory signatures. In this regard, MEG is an appealing research tool as it has both an excellent temporal (and thus spectral) resolution, and it allows for a reliable source reconstruction (relative to EEG) with a reasonable spatial resolution (Hansen et al., 2010).

In line with the advent of producing SL experiences in the lab, the participants employed in the present study are long-term mindfulness meditation practitioners. Mindfulness is defined and practiced as a non-judgmental awareness of bodily or mental experiences arising in the present moment. Regardless of how pleasant or unpleasant the arising experiences are, the meditator trains not to cling to, nor to push them away, but rather to treat them with acceptance, openness and curiosity, watching them arise, play in the theater of the mind and finally dissolve back into the space of the mind (Kabat-Zinn, 1990). Mindfulness is a current and widespread form of Buddhist practice (Williams and Kabat-Zinn, 2011), and has been shown to enhance cognitive functions such as attentional abilities, emotional regulation, executive functions and memory (Chiesa and Serretti, 2010; Chiesa et al., 2011), altering the brain circuits and neuropsychological mechanisms underlying these functions (Cahn and Polich, 2006; Davidson and Lutz, 2008; Lutz et al., 2008; Hölzel et al., 2011b), and even altering brain structure in regions typically activated during mindfulness meditation (Lazar et al., 2005; Hölzel et al., 2008, 2011a).

**Figure 1:**
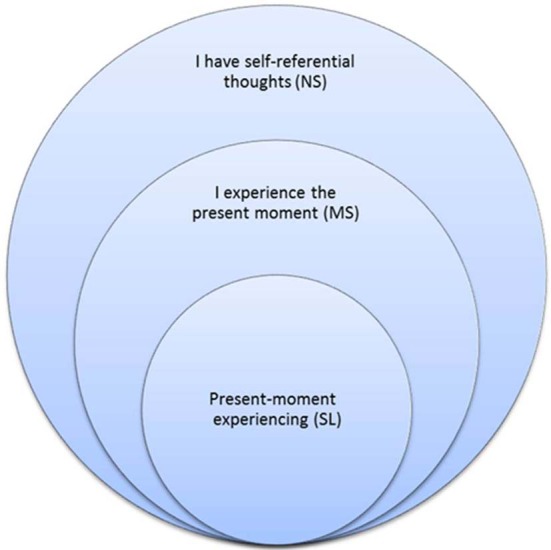
**Working model of self-awareness modes.** NS, MS and SL as encapsulated processing modes.

One of mindfulness's mechanisms of action is an altered sense of self (Hölzel et al., 2011b). Mindful awareness induces a sharper sense of the normally perceived subjective sense of self (Lutz et al., 2008), but treats it as an object of meditation. This cultivated shift to an “observer perspective” (Kerr et al., 2011) induces a change in the perspective of self and first-person experience. Indeed, a recent integrative theoretical framework and systems-based neurobiological model suggests understanding mindfulness by focusing on self-processing and the neural networks underlying self-awareness, self-regulation, and self-transcendence (Vago and Silbersweig, 2012). A growing number of studies show that mindfulness alters DMN and self-related activity and connectivity (EEG: Berkovich-Ohana et al., 2012; Lehmann et al., 2012; fMRI: Pagnoni et al., 2008; Brewer et al., 2011; Ives-Deliperi et al., 2011; Froeliger et al., 2012; Hasenkamp and Barsalou, 2012; Taylor et al., 2013). In particular, Farb et al. (2007) used fMRI and a mindfulness-based stress reduction (MBSR, Kabat-Zinn, 1982; Kabat-Zinn et al., 1992) intervention to dissociate narrative from experiential modes of processing. The present study continues Farb et al. (2007) in using mindfulness meditators for revealing the neural correlates of momentary (parallel to MS) and across-time (parallel to NS) self processing, but goes further in exploring SL: momentary phenomenal experience free of the sense of agency and ownership. Figure 1 illustrates the encapsulated relationship between NS, MS and SL.

The working basis for the present study's design is that long-term mindfulness meditators: (1) are adept in keeping their attention for extended time periods on an object of choice, be it a physical object, the breath, or a produced state of self; and (2) develop through practice their goal of dissolving the experience of a fixed subjective core comprising their self-identity. Thus, such participants were recruited and requested to mentally project states representing NS, MS, and SL, while their brain activity was recorded by MEG. The purpose of the study is to map the differential neural activity related to NS and MS, as well as characterize SL, a present-centered conscious experience devoid of an experiencing subjective self. The study's aims and hypotheses are to:

1. *Map the neural correlates of NS*. Given that MS is at play also during NS, contrasting the two conditions is expected to reflect NS attenuation, and is hypothesized to result in a reduction of mPFC high-gamma oscillatory activity. This part of the study is expected to bridge results from the prevalent fMRI imaging literature and the present MEG methodology.
2. *Map the neural correlates of MS*. Given that both MS and SL share a present-centered experiential aspect, differing only in terms of the experiencer—the agency/ownership aspects accompanying experience, contrasting MS and SL is expected to reflect the neural correlates of MS attenuation. Here predictions are less clear, nevertheless, the IPL and insula are likely to play roles. Localizing this differential activation within the frequency domain will be a novel contribution of the present study.
3. *Use first-person reports for grouping the MEG data* and identifying the neural correlates of the subjective aspects of the Buddhist-described “selfless” experience.

## Materials and methods

### Participants

Sixteen experienced meditation practitioners were recruited for the research project. Two participants' data were discarded early on in the experiment. The first due to complaints of tiredness and lack of focus, and the second due to back pain (to the point of stopping the MEG recording). In addition, in order to establish a common frame of reference for the first-person descriptions, as well as control for confounds that might result from different sources of training, only participants practicing very similar forms of meditation were included in the study. Thus, the data of two further participants (Zen and non-dual practitioners, see Lutz et al., 2007 for details on these forms of meditation) were not analyzed. The remaining 12 practitioners, all mindfulness meditators practicing similar forms of Vipassana, either originating from or inspired by the Buddhist Theravada tradition, comprise the participants of the present study. All are right-handed (9 males and 3 females, mean age 45.2, *SD* = 11.3, ranging from 31 to 66) with no history of mental or neurological disease. All of the participants are long-term practitioners with an average of 16.5 (*SD* = 7.9, ranging from 9 to 34) years of meditation practice, and an average of 11,225 (*SD* = 9909, ranging from 1290 to 29,293) total hours of meditation practice. All the performed procedures are in compliance with the Code of Ethics of the World Medical Association (Declaration of Helsinki), and were approved by the Research Ethics Board of Bar-Ilan University. The participants gave their written consent and were financially compensated for their time.

### Pre-recording procedures

The participants were welcomed and introduced to the experiment and the research facility. They then filled out forms noting their agreement to participate in the experiment, their personal details, and a form estimating their formal meditation experience. Pre-task procedures included an average of 45 min of clarifying the study's setup, tasks and stimuli using a PowerPoint presentation and allowing time for questions and discussion. In this way, misunderstanding, alteration, or rejection of the scripts provided by the researchers, were minimized (Roepstorff, 2001).

### Tasks

The experiment included seven experimental sessions. Of these, only the “self” session (fifth in order) is reported here. Each session consisted of performing tasks during which the participant's brain activity was recorded. This was followed by an interview conducted via the intercom system, during which brain activity was not recorded. The participants were encouraged to stretch their limbs and relax during the interview, but were requested not to move and to keep their eyes closed while performing the tasks. To correct for head and body movements during the interview session, head-shapes were re-registered at the beginning of each session. A 20-min break was suggested to the participants after completing the 5th session of the experiment, during which refreshments were offered. Total time in the MEG ranged from 2 to 3 h. The participants' condition was closely monitored throughout the experiment via the intercom (during the interview sessions) and the closed-circuit TV camera (at all times). In addition, participants were asked a number of times throughout the experiment if they were tired and needed an additional break.

The task relevant to the present study is the “self task.” Participants were requested to mentally project themselves into certain self-related states, which had been described and discussed during the PowerPoint presentation. The session included 3 conditions, each repeated 3 times in succession for 30 s. This more ecologically-valid design (in contrast to the commonly employed event-related designs) was chosen due to mediators' heightened capacities in directing and sustaining attention (Brefczynski-Lewis et al., 2007; Lutz et al., 2008). The first 4 s of each 30-s epoch were omitted so as to allow participants sufficient time to enter the states (SL in particular). This decision was made after consulting a well-known, very-long-term, meditation teacher, concerning the study design, and after 2 pilot runs (with two of the authors, Aviva Berkovich-Ohana and Yair Dor-Ziderman, who are also long-term meditators). A recording with instructions for each condition was sounded, after which the participant performed the requested task. At the end of the 30 s, a sound was heard indicating to the participant to stop and rate task performance success (on a 1–3 scale). This measure was incorporated in order to allow *post-hoc* identification of bad trials (button press of 1). However, as none of the participants in any of the conditions reported here indicated such bad trials, this measure was not further used. After pressing the corresponding button, the next instruction was delivered. The session was followed by a structured interview conducted via the intercom system.

The exact instructions for each self-projected condition were:

- “narrative” condition (NS)—*“Try to think what characterizes you*.”
- “minimal” condition (MS)—*“Try to experience what is happening to you* at the present moment.”
- “selfless” condition (SL)—*“Try to experience what is happening at the present moment, when you are not* in the center.”

### Data acquisition

#### MEG

MEG recordings were conducted with a whole-head, 248-channel magnetometer array (4-D Neuroimaging, Magnes 3600 WH) in a magnetically-shielded room. Reference coils located approximately 30 cm above the head oriented by the *x*, *y*, and *z* axes were used to remove environmental noise. Head position was indicated by attaching 5 coils to the scalp and determining, to a 1 mm resolution, their position relative to the sensor array before and after measurement. Head localization was performed before and after each set of tasks to determine degree of head movement. Head shape and coil position were digitized using a Pollhemus FASTTRAK digitizer. Brain signals were recorded with a sample rate of 1017.25 Hz and an analog online 0.1–400 Hz band-pass filter. The instructions for each condition were presented using E-prime 1.0 and delivered via a STAX SRS-005 amplifier and SR-003 push-pull electrostatic ear speakers coupled by a vinyl tube to silicon earpieces to prevent magnetic noise within the shielded room. Task performance ratings were collected using a LUMItouch photon control response box.

#### Subjective reports

***Retrospective reports.*** Participants were asked to provide retrospective reports regarding their perceived (relative to past experiences) success and stability (on a 1–10 scale, with 1 denoting “very low” and 10 denoting “very high”) in performing the tasks, as well as report on the emotional content of their experiences during the different tasks.

***Introspective reports.*** Participants were asked to describe their SL experience freely and in their own words, without reflection or judgment (Jack and Roepstorff, 2002; Schooler, 2002; Lutz and Thompson, 2003). In addition, the descriptions were collected immediately after they were produced in order to minimize reliance on episodic recall (Jack and Roepstorff, 2002).

### MEG data analysis

#### Cleaning and preprocessing

Data processing and analysis was performed using Matlab® R2009b and FieldTrip toolbox for MEG analysis (Open Source Software for Advanced Analysis of MEG, Oostenveld et al., 2011). Data were cleaned for line frequency (by recording on an additional channel the 50 Hz from the power outlet, and subtracting the average power-line response from every MEG sensor), and 24 Hz building vibration (measured in *x*, *y*, and *z* directions using 3 Bruel and Kjaer accelerometers) artifacts (Tal and Abeles, 2013). The data from the 3 “self” tasks were then segmented into non-overlapping 2-s epochs. Each epoch was visually examined for muscle and jump (in the MEG sensors) artifacts. Contaminated epochs were discarded. No malfunctioning MEG sensors were identified. To ensure the removal of all heartbeat, eye, and muscle artifact, an independent component analysis (ICA) was performed on the data (Jung et al., 2000). Segmented data were down-sampled to 339 (1017/3) Hz to speed up data decomposition. The data were then decomposed into a set of independent components (248, as the number of sensors) ordered by degree of their explained variance. Components indicating heartbeats or eye movements were determined from a visual inspection of the 2D scalp maps and time course of each component. 2.6 ± 1.2 components were taken out on average, and the remaining components were then used to reconstruct the pre down-sampled data.

#### Sensor-level analyses

In order to level the number of trials for all participants and conditions, the first 32 of the remaining epochs were marked as the data for further sensor-level analyses. The segmented 2-s epochs were multiplied by a Hanning taper, and subjected to a Fast Fourier Transformation (FFT) for the frequencies ranging from 0.5 to 100 Hz. This resulted in a power spectrum with a frequency resolution of 0.5 Hz for each epoch. The power spectra were then averaged across the epochs of each condition, thus obtaining the mean power for each condition and participant. The next step involved calculating, for each frequency of each sensor of each participant, a power percent-in-signal-change (PSC) metric, for estimating power differences in NS vs. MS and MS vs. SL. PSC was computed in the following manner: for sensor *S*, frequency *f*, and power values of conditions *A* and *B*, PSC[*S*(*f*)] = [(*A/B*) − 1] if *A* > = *B*, and [1-(*B*/*A*)] if *B* > *A*. This manipulation yields a balanced PSC distribution centered on 0. Each participant's PSC values for the two comparisons were then collapsed across all sensors, and averaged across the delta (0.5–3.5 Hz), theta (4–7.5 Hz), alpha (8–12.5 Hz), beta (13–25 Hz), gamma (25.5–59.5 Hz), high-gamma (60–80 Hz), and very-high-gamma (80.5–100 Hz) frequency bands. To reduce dimensionality prior to localization, 1-sample *t*-tests were performed for each frequency band and for each comparison against the null hypothesis that the PSC measures came from a continuous, normal distribution with a zero mean. Results were then Bonferroni-corrected. Finally, 2D scalp topographies of the mean PSC in the significant frequency bands and comparisons were created.

#### Source-space projection

Localization was performed for the frequency bands which evidenced significant PSC in the sensor-level data. Sources were estimated using Synthetic Aperture Magnetometry (SAM, Robinson and Vrba, 1999). SAM is an adaptive nonlinear minimum-variance beamformer algorithm. It calculates the signal covariance from the MEG sensor data and uses it in conjunction with a forward solution for the dipoles at each 3D brain voxel (of a specified size) to construct optimum spatial filters. The spatial filtering suppresses interference of unwanted signals from other locations.

For source estimation, the pre-ICA data were used. A number of works have shown that interfering biomagnetic sources such as cardiac, respiratory, and eye movements are effectively suppressed by beamforming (e.g., Sekihara et al., 2006; Brookes et al., 2008, 2011). Data were band filtered (using the SAM default IIR filter) for each participant and condition in the frequency bands specified through the sensor-level analysis. Covariance matrices, and subsequently SAM weights, were computed for each 5 cubic-mm voxel using the data from the two conditions participating in each signal change calculation, for each frequency-band-filtered time-series data. For each voxel, the data were multiplied by the weights, thus creating “virtual sensor” time-series, which were then transformed via FFT to the frequency domain, thus deriving power values. Finally, PSC values (same metric as the one described in the sensor-level analysis section, pseudo-F in SAM) were computed for each comparison, participant and each and every voxel.

To facilitate group analysis, head models were constructed by co-registering each participant's SAM volume to a previously obtained MRI scan (T1-weighted anatomical images acquired with high-resolution 1-mm slice thickness, obtained by one of the authors (Aviva Berkovich-Ohana) by means of a 3T Trio Magnetom Siemens scanner located at the Weizmann Institute of Science, Rehovot, Israel) based on the position of the fiduciary markers established during the digitization phase. Each participant's MRI image and its co-registered SAM volume were then transposed into a common anatomical space (Talairach coordinates, Talairach and Tournoux, 1988). Voxel-level group statistics, for each comparison and frequency band, were conducted using a non-parametric permutation analysis procedure (2000 permutations, Nichols and Holmes, 2001; Singh et al., 2003), and corrected for multiple comparisons based on a Monte Carlo simulation of random noise distribution (using AFNI's 3dClustSim module, Forman et al., 1995).

### Neurophenomenological analysis

#### Subjective reports

***Success and stability.*** For assessing whether participants' ratings for perceived success and stability were different for the different tasks, repeated-measures ANOVA was conducted for success and stability as dependent variables.

***Emotional content.*** Participant reports of emotional content during each task were collected and arranged in 4 categories: *neutral*—here participants either reported no emotional content or explicitly stated a neutral state; *positive*—here participants reported only positive emotions (such as enjoyment, comfort, quiet, pleasant, rest, and lightness); *negative*—here participants reported only negative emotions (such as pride, fear, anxiety, confusion, insecurity, and dislike), and mixed—which included reports of both positive and negative emotions. In addition, a number of participants spontaneously reported low level of emotions (NS—4 participants, 2 in the *negative* category and 2 in the *mixed* category; SL—1 participant in the *mixed* category). A note regarding the categorization of “pride” as a negative emotion: this is in alignment with the Buddhist context (Goleman, 1995; Chambers et al., 2009), given that the participants are long-term practitioners of Buddhist traditions.

***Meditation experience.*** Meditation experience was gauged using a normalized measure incorporating both total number of years and hours of meditation. The maximum values of meditation year and hour estimates were extracted, and all other values were divided by them, resulting in values between 0 and 1. The two metrics were then averaged, giving equal weight to meditation years and hours.

***First-person SL descriptions.*** A careful reading by the authors of the participants' first-person descriptions of their SL experiences indicated three rather broad but distinct types of experiences. Age was ruled out as a confounding factor [ANOVA, *F*_(1, 11)_ = 3.76, *ns*]. The suggested categorization was further validated by presenting the raw participant descriptions and category explanations (as presented below but without the examples) to 12 naïve referees (graduate students and postdoctoral researchers), and asking them to categorize the descriptions according to the suggested scheme. Descriptions which were categorized differently than the suggested scheme by more than one referee were excluded from the analysis. Two descriptions were thus removed (sub14 and sub16's, 4 referees categorized each of them differently), resulting in 10 SL descriptions. The participants' descriptions (including those finally excluded from the analysis) and their categorization are presented below in Table 1. The suggested categories are:

**Table 1:**
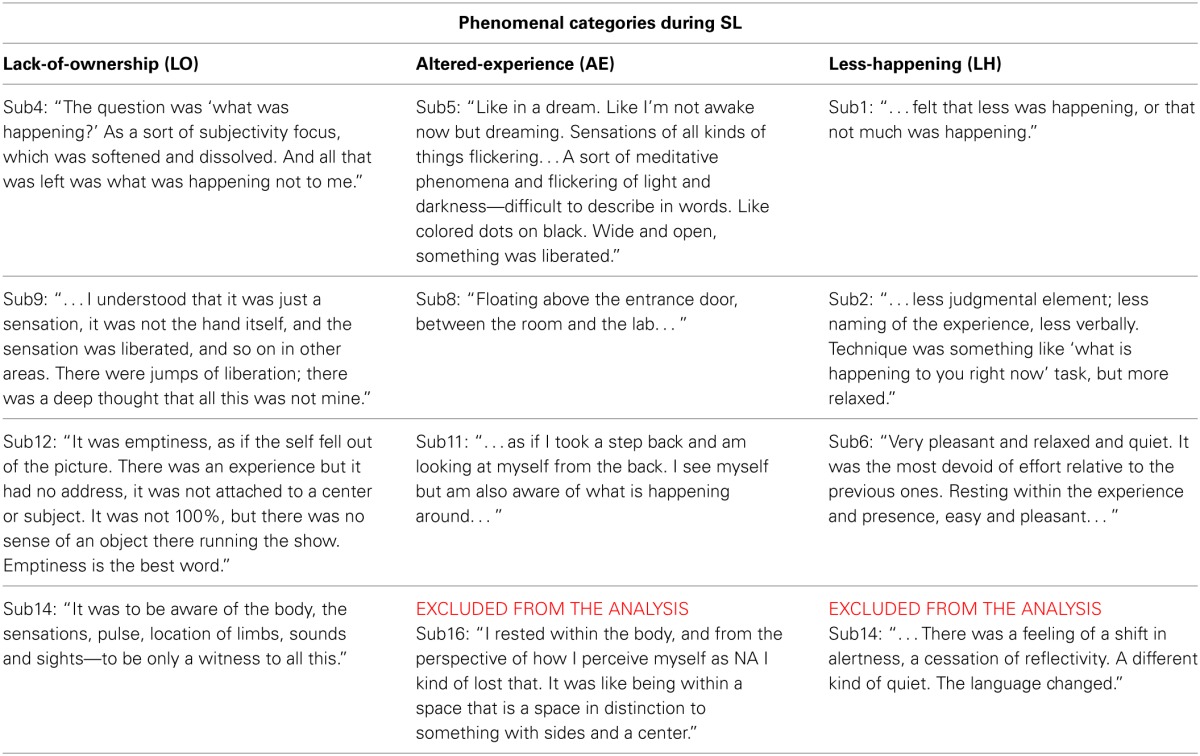
**SL descriptions and their phenomenological categorization**.

Participants' first-person SL experiences descriptions arranged by the suggested categorization scheme. Left column = LO category; middle column = AE category; right column = LH category. Descriptions excluded from the analysis due to the validation process are so noted.

- Lack-of-ownership (LO): The 4 participants in this category reported experiencing what was happening, only with the sense of agency/ownership absent. As an example, participant sub12 described: *“It was emptiness, as if the self fell out of the picture. There was an experience but it had no address, it was not attached to a center or subject … ”*
- Altered-experience (AE): The 4 participants in this category reported an altered experience of their bodies/senses/spatial-context. For example, sub11 reported: *“On the level of feeling and sensing—as if I took a step back and am looking at myself from the back. I see myself but am also aware of what is happening around … ”*
- Less-happening (LH): The grouping of this category was somewhat looser than the other categories. The 4 participants in this category reported a quieting or general relaxation of body, reflectivity, cognition, or experience in general. For example, participant sub6 reported: *“Very pleasant and relaxed and quiet. It was the most devoid of effort relative to the previous one …* ”

#### MEG source estimates

To identify within the MS vs. SL beta-band (the only frequency band relevant for characterizing SL—see results section) network regions specifically correlated with the phenomenological categories, MEG source estimates of each phenomenological category (vs. the other two categories) were derived in a manner similar to the one described above. Group statistics, limited to the network of interest (significant MS vs. SL beta band regions), were computed on the Talairach-transformed individual images using non-parametric random permutations with a 2-sample *t*-test statistic.

## Results

### Subjective reports

The first-person reports indicated that the participants were able to successfully produce the different self-states. The means for *task success* were high (on a 1–10 scale with 1 denoting “very low” and 10 denoting “very high”) for the NS, MS, and SL tasks (8.6 ± 0.9, 7.8 ± 1.4, and 8 ± 1.2, respectively). In addition, participants reported high measures of *task stability*: 8.2 ± 1.6, 8 ± 1.4, and 7.6 ± 1.5 for NS, MS, and SL, respectively. These indicate that the participants managed—in their subjective experience—to produce and maintain the requested self-states in a stable manner for the task duration. The ANOVA indicated no significant differences between the states for both *task success* [*F*_(2, 11)_ = 2.2, MSE = 1.58, *ns*] and *task stability* [*F*_(2, 11)_ = 1.08, MSE = 1.39, *ns*]. These results help rule out attribution of between-conditions differences to task difficulty.

**Figure 2:**
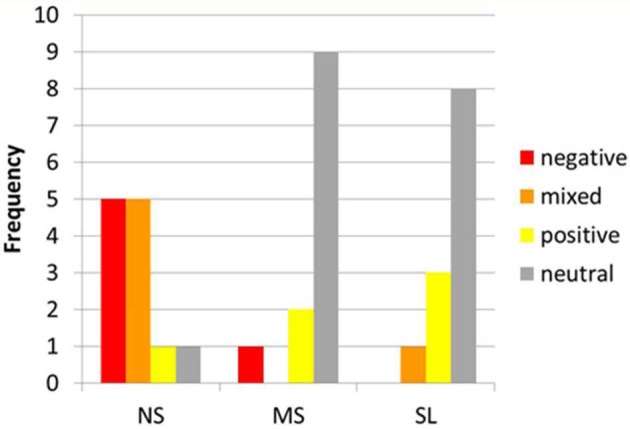
**Emotional content during NS, MS, and SL.** Distribution of emotional content among participants (*x*-axis) during NS, MS, and SL (*y*-axis). Note the marked difference between NS and other 2 conditions regarding negative and mixed vs. neutral emotions.

The emotional content reported by the participants did differ between conditions. The emotional profiles during each of the tasks are depicted in Figure 2. A marked difference can be observed between the NS and the other two self-tasks. While in the NS condition 10 participants reported negative (5) or mixed (5) emotions, in the MS and SL conditions only one participant reported negative or mixed emotions, and on the other hand, 8 (SL) and 9 (MS) reported a neutral affective state, while the remaining 3 and 2 (respectively) participants reported positive emotions.

### Sensor-level results

**Figure 3:**
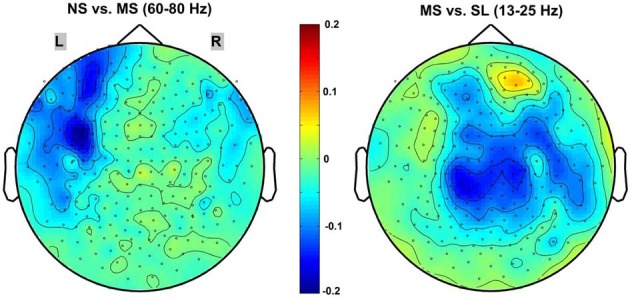
**2D scalp maps of frequency bands with significant power PSC.** 2D topographic representations of significant sensor-level power PSC for the NS vs. MS high-gamma 60–80 Hz (**left**), and MS vs. SL beta 13–25 Hz (**right**). Dots on the map represent sensors; color bar scale indicates PSC from 0.2 (dark red) to −0.2 (dark blue).

Of the frequency bands tested (delta, theta, alpha, beta, gamma, high-gamma, very-high-gamma), the sensor level results indicated a significant decrease in global (all 248 sensors) PSC between the NS and MS conditions only in the high gamma 60–80 Hz band (mean PSC = −0.052 ± 0.0472; *p* < 0.02, 1-sample *t*-test, Bonferroni-corrected). In contrast, the only frequency band evidencing a significant PSC when contrasting the MS and SL conditions was the 13–25 Hz beta band (mean PSC = −0.103 ± 0.1107; *p* < 0.05, 1-sample *t*-test, Bonferroni-corrected). The other frequency bands evidenced no significant power PSC differences—even prior to the Bonferroni correction. Figure 3 provides 2D topographic representations of the sensor level power PSC in these two significant frequency bands. Note the different topography for the two comparisons, with the high-gamma NS vs. MS decreases in power occurring predominantly over frontal-left electrodes and the beta MS vs. SL decreases being more central and right lateralized.

### Source localization estimates

SAM beamforming source estimates are reported for the 60–80 Hz high-gamma band for NS vs. MS, and in the 13–25 Hz beta band for MS vs. SL—as indicated by the sensor-level data. As a comparative measure, the complementing high-gamma (for MS vs. SL) and beta (for NS vs. MS) localization solutions are also reported.

#### NS vs. MS source estimates

**Figure 4:**
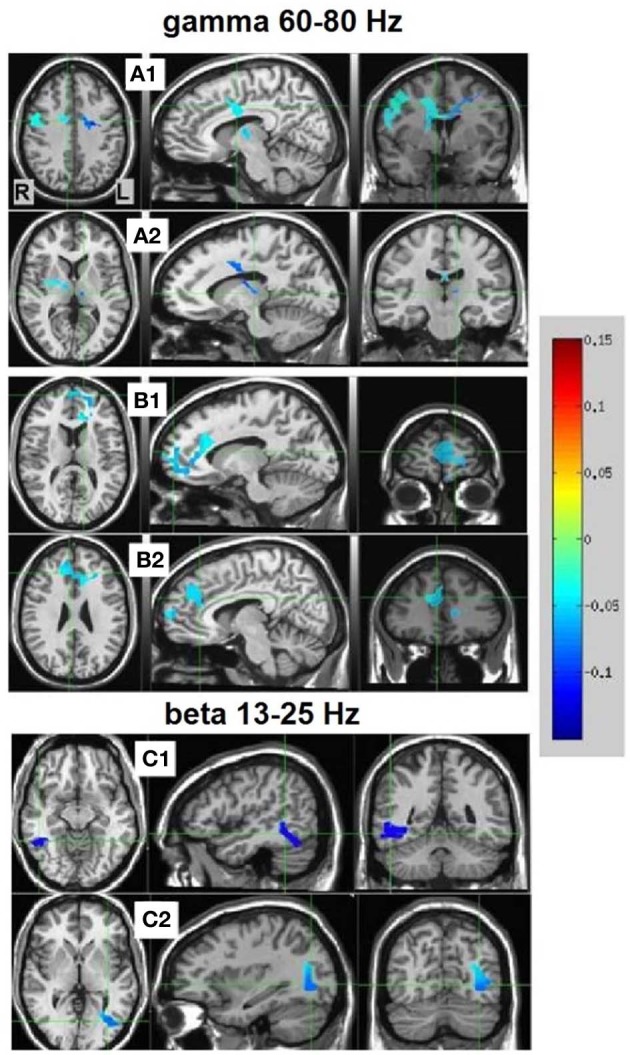
**NS vs. MS beamforming source estimates in the gamma (60–80 Hz) and beta (13–25 Hz) frequency bands.** Axial, sagittal, and coronal views (left to right) of group (*n* = 12) PSC source estimates overlayed on the Colin template. Note that in all images right and left sides are crossed. Color bar indicates PSC degree. Gamma band clusters: Cluster A is presented in 2 views. Crosshairs in **(A1)** are on the right medial anterior cingulate, and in **(A2)** on the left thalamus. Cluster B is presented in 2 views. Crosshairs in **(B1)** are on the left anterior cingulate, and in **(B2)** on the right anterior medial prefrontal cortex. Beta band clusters: Crosshairs in **(C1)** are on the right fusiform gyrus, and in **(C2)** on the left middle occipital gyrus.

Resulting NS vs. MS 60–80 Hz high-gamma band images were thresholded at the maximum *t*-value possible for a non-parametric random permutation analysis with 2000 permutations (*t* = 4.863, see methods section for details), yielding 2 robust (*p* < 0.0005, corrected) rather large clusters (314 and 264 voxels) spanning almost exclusively frontal regions, and all indicating decreases in gamma power in MS relative to NS (reflecting NS attenuation, see introduction). The larger cluster (mean PSC −0.083, **A1** and **A2** in Figure 4) was more posterior, including right and left precentral gyrus, middle cingulate cortex, middle frontal gyrus and thalamic regions. In the right hemisphere, the cluster included the posterior part of the inferior frontal gyrus and operculum, lentiform nucleus and caudate body. Two thirds of the cluster was in the right hemisphere; however, the left hemisphere PSC was stronger. The second cluster (264 voxels, mean PSC −0.086, **B1** and **B2** in Figure 4), was more anterior (prefrontal) and mostly left-lateralized (76%). The cluster spanned bilateral dorsal and anterior regions of the medial frontal gyrus, superior frontal gyrus, and dorsal ACC. More ventrally, left-lateralized regions included subgenual ACC, mid orbital gyrus, middle frontal gyrus and middle cingulate cortex. See Figure 4 and Table 2 for more details and a visual depiction.

**Table 2:**
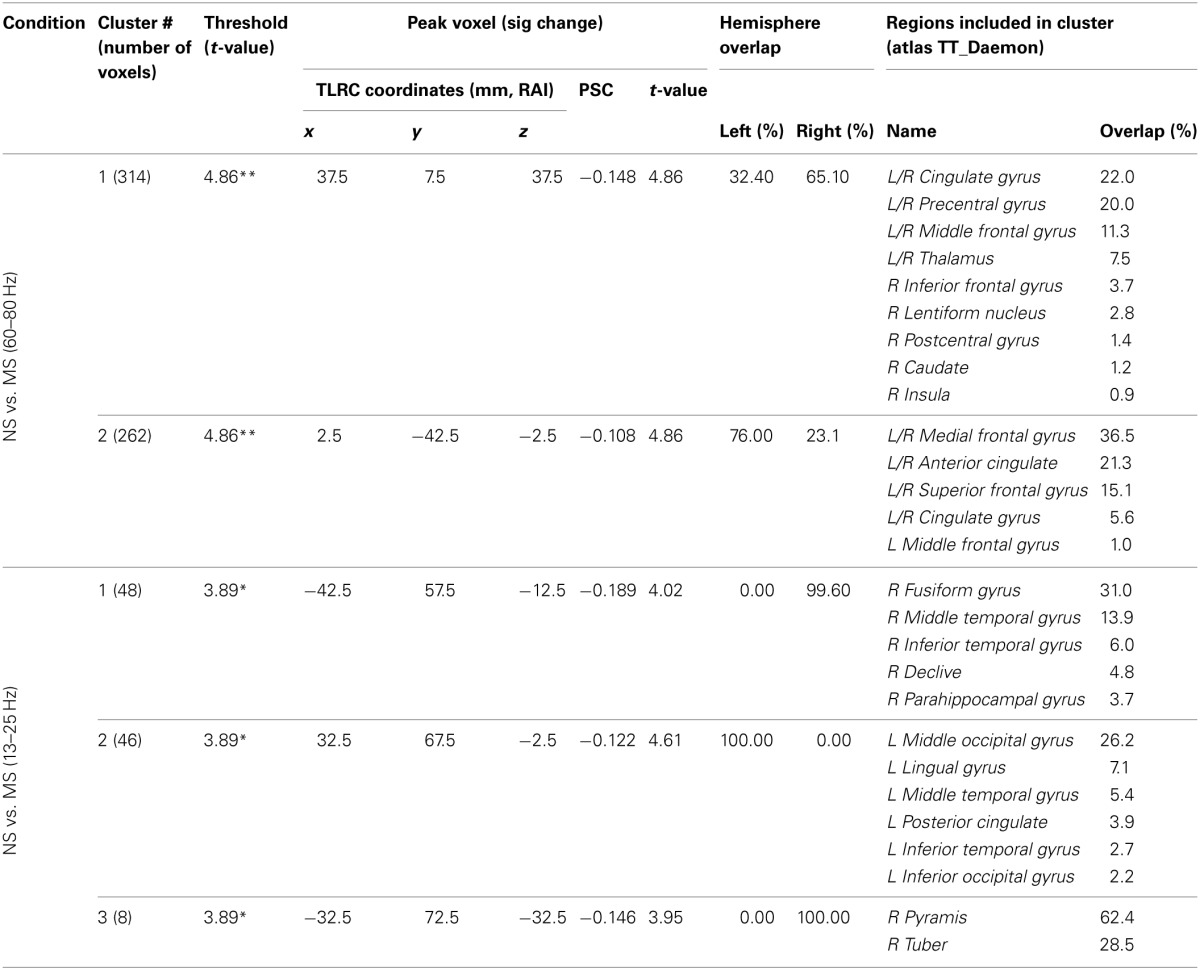
**NS vs. MS beamforming solutions for gamma (60–80 Hz) and beta (13–25 Hz)**.

Information supplied includes number of voxels in each cluster, thresholds, peak voxel characteristics, hemispheric overlap, and brain regions included in the cluster. The Afni supplied TT Daemon atlas was used. Due to poor resolution and signal leakage to non-brain regions, overlap percentages do not always add up to 100%. ^*^p < 0.0025; ^**^p < 0.0005 (corrected).

In the beta band (13–25 Hz), the NS vs. MS contrast also resulted in significant PSC results, albeit markedly less robust compared to the gamma band results and solely in posterior regions. These images were thresholded at *t* = 3.887, yielding 3 significant clusters at the *p* < 0.0025 (corrected) level. Right-hemisphere regions included mainly the fusiform and middle temporal gyrus (48 voxels, mean PSC −0.148, **C1** in Figure 4), and a small cluster in the right cerebellum (8 voxels, mean PSC -0.141, not shown). Left-hemisphere regions encompassed mainly the middle occipital and lingual gyrus (36 voxels, mean PSC −0.099, **C2** in Figure 4). See Figure 4 and Table 2 for more detail and visual depiction.

#### MS vs. SL source estimates

**Figure 5:**
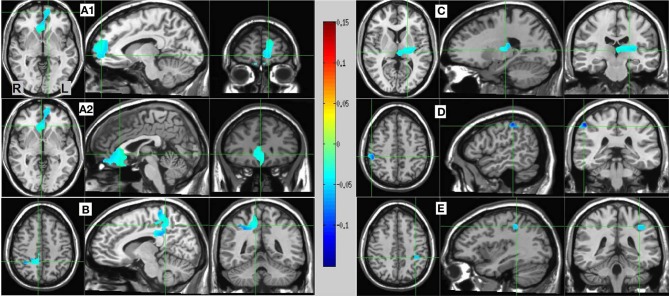
**MS vs. SL beamforming source estimates in the beta (13–25 Hz) band.** Axial, sagittal, and coronal views (left to right) of group (*N* = 12) SAM pseudo-*F* source estimates overlayed on the Colin template. Note that in all images right and left sides are crossed. Color bar indicates PSC degree. Cluster A reveals prefrontal deactivations in two views: the crosshairs in **(A1)** are on the left anterior medial prefrontal gyrus, and in **(A2)** on the right subgenual anterior cingulate. Cluster **(B)** shows deactivation in the posterior medial cortex, with the crosshairs pinpointing the right precuneus. Cluster **(C)** shows deactivation in the left thalamus; and clusters **(D)** and **(E)** deactivations in the right and left inferior parietal lobules, respectively.

Resulting MS vs. SL 13–25 Hz beta band source estimates were thresholded at *t* = 3.887, yielding six clusters (for a total of 390 voxels) significant at *p* < 0.0025 (corrected), all indicating decreased beta power in SL relative to MS (reflecting MS attenuation, see introduction). No significant clusters were found in the high-gamma band range. The largest cluster (182 voxels, mean PSC −0.067, **A1** and **A2** in Figure 5) consisted of prefrontal and left lateralized regions including the superior frontal gyrus, ventral mPFC, and rostral ACC; bilateral regions including the subgenual ACC, mid orbital, and rectal gyrus; and a region located in the right middle frontal gyrus. The 2nd cluster (109 voxels, mean PSC −0.08, **B** in Figure 5) was completely in the right hemisphere and included regions from the postcentral gyrus, middle cingulate cortex, paracentral lobule, precuneus, and posterior cingulate cortex (PCC), and the IPL. The 3rd cluster (66 voxels, mean PSC −0.072, **C** in Figure 5) included large portions of the left thalamus and lentiform nucleus, extending medially to the left posterior insula. The 4th cluster (13 voxels, −0.1 PSC, **D** in Figure 5) was more anterior and lateral compared to the 2nd cluster, and included regions of the right IPL and postcentral gyrus. The 5th cluster (11 voxels, −0.081 PSC, **D** in Figure 5) was located in the left IPL, and the 6th cluster (9 voxels, −0.105 PSC, not shown) in the right precentral gyrus and the posterior middle frontal gyrus. See Figure 5 and Table 3 for more details and a visual depiction.

**Table 3:**
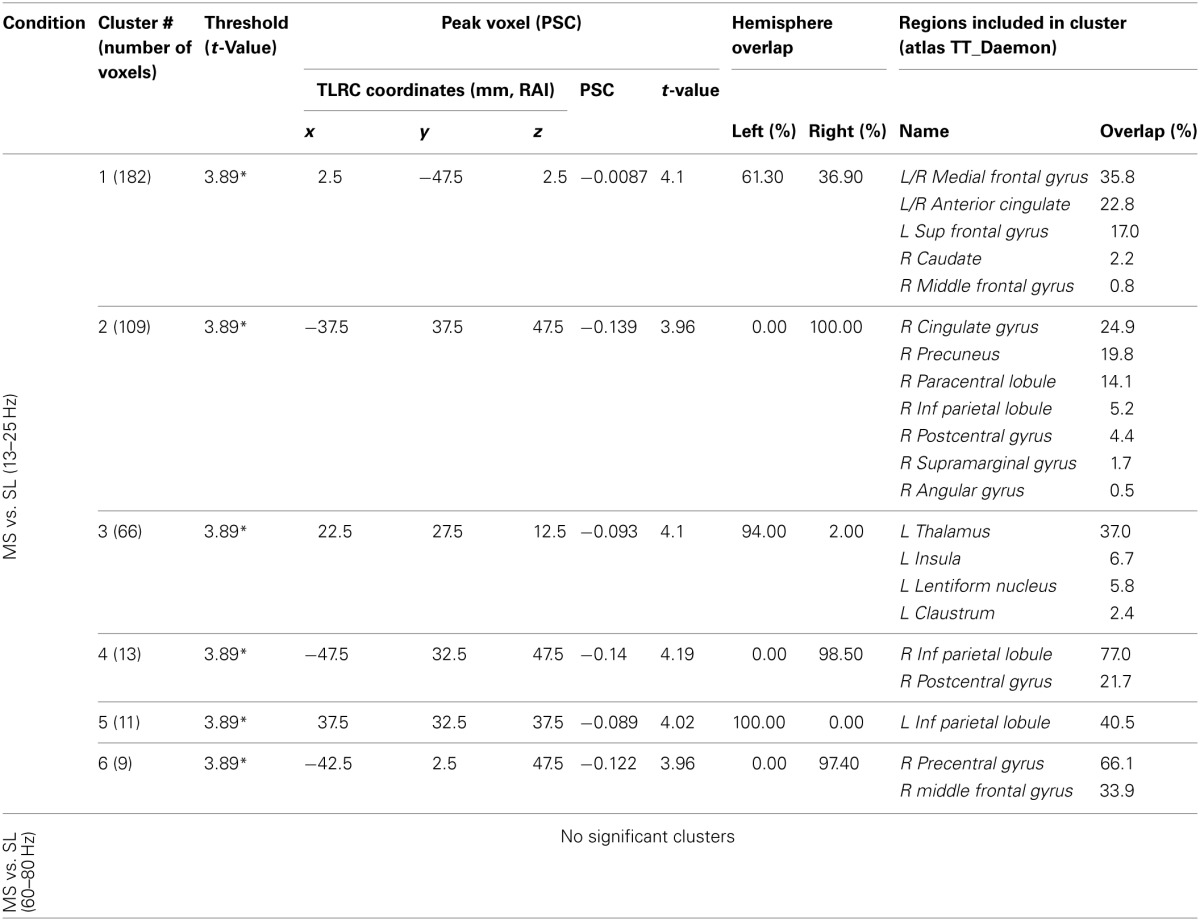
**MS vs. SL beamforming solutions for gamma (60–80 Hz) and beta (13–25 Hz)**.

Information supplied includes number of voxels in each cluster, thresholds, peak voxel characteristics, hemispheric overlap, and brain regions included in the cluster. The Afni supplied TT Daemon atlas was used. Due to poor resolution and signal leakage to non-brain regions, overlap percentages do not always add up to 100%. ^*^p < 0.0025 (corrected).

### Neurophenomenology

**Figure 6:**
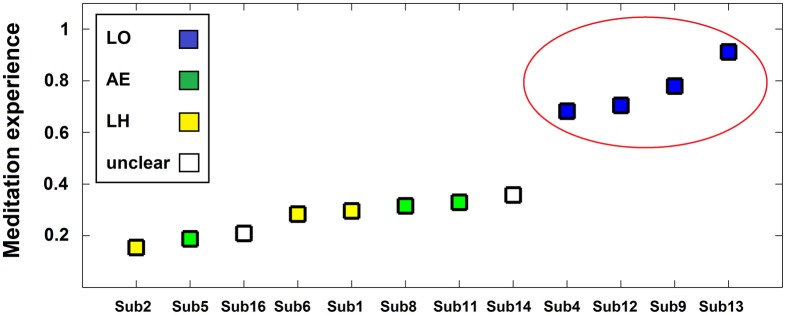
**Phenomenological categories and meditative expertise chart.** Participants (*x*-axis) are plotted as a function of meditative expertise (*y*-axis) from least to most -experienced. The meditation experience measure is a normalized (0–1) measure incorporating both years and hours of meditation practice. Colors indicate phenomenological category of participants' SL description (blue = LO [Lack-of-ownership], green = AE [Altered-experience], yellow = LH [Less-happening], white = unclear). Note the increase in meditative expertise for the LO group (circled in red).

The neurophenomenological data consisted of the participants' first-person descriptions of their SL experiences, and the MS vs. SL beta-band network described above. The phenomenal categorization yielded three categories (LO, AE, and LH, as detailed in the methods section). The LO category participants were also the most experienced and formed a distinct group with meditation experience being qualitatively higher than the members of the other categories (see Figure 6). The other two experience categories were mixed in terms of their members' meditative experience.

**Figure 7:**
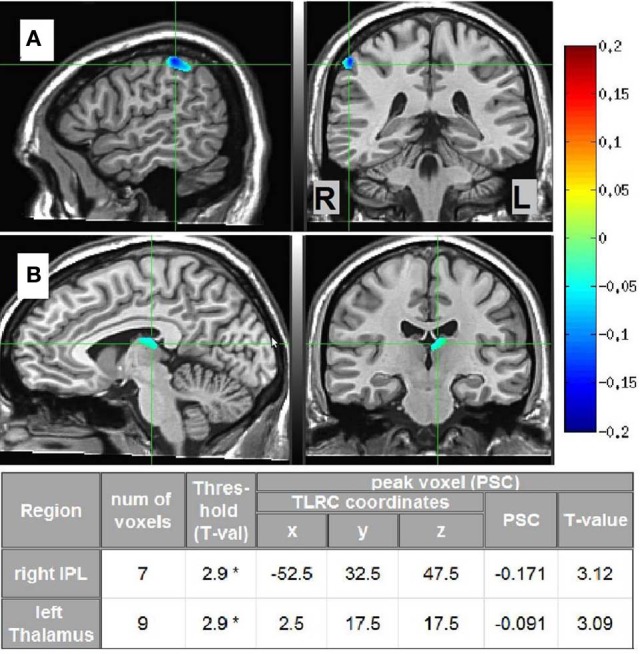
**LO source estimates.** Sagittal and coronal views of significant (^*^p < 0.02, corrected) LO source estimates (*n* = 4) relative to the other participants (*n* = 6, not including “unclear” category), overlayed on the Colin template. Crosshairs are in **(A)** on the right IPL; and in **(B)** on the left dorsomedial thalamus. The table provides Talairach coordinates, PSC, and other cluster details.

Source estimates characterizing each phenomenal category were obtained by pitting the beta-band MS vs. SL images of the participants in each phenomenological category against the other two categories. Two significant cluster were found, distinguishing LO participants (*n* = 4) from the others (*n* = 6, not including “unclear” category). The first cluster was located in the right IPL and the second in the left dorsomedial thalamus. The other phenomenological categories did not yield any significant clusters. In addition, the same analysis but within the gamma-band NS vs. MS images yielded no significant source estimates for any of the phenomenal categories. Thus, methodological triangulation (Gallagher, 2002; Jack and Roepstorff, 2002) of distinct phenomenology, meditative expertise and SAM source estimates in the beta band, culminated in a distinct neurophenomenological characterization of the LO category. Visual depictions of these regions, along with cluster details are presented in Figure 7.

### Issues of validity

#### Myogenic artifact

As participants reported a more neutral emotional state during MS relative to NS, it might be argued that the reported decrease in high-gamma power (in which myogenic artifacts may manifest) reflects a more relaxed state and thus reduced muscle-activity, and not a difference in neural activity. There are, however, a number of arguments that make this unlikely: (1) Muscle artifacts are less of a problem in MEG relative to EEG measurements, partly due to the possibility of reliably localizing the effects' activation locus. In fact, MEG has been previously employed for determining whether EEG high frequency components may have a muscular origin (Zimmermann and Scharein, 2004; Claus et al., 2012). (2) Pre-emptive measures were taken to counter muscle artifacts during both data collection (supine positioning, eyes closed) and data cleaning (visual inspection of all the data). (3) The high-gamma effect found here is dissimilar to typical myogenic artifacts (described in Muthukumaraswamy, 2013) in several ways. The effect, on the sensor level is: (a) highly lateralized (and it is unlikely that for all the participants the artifact was confined to one hemisphere); (b) does not extend to the montage borders (which is the normal case for muscle artifacts); and (c) the activity is narrowly confined to the 60–80 Hz band (while myogenic artifacts tend to be “patchy” and to command a wider spectrum). (4) Finally, the reported regions are consistent with a well-established body of literature.

In addition, in order to empirically test the link between increased emotionality, the myogenic artifact and increased high-gamma PSC, a further analysis was conducted. The values of the peak activation voxels of each of the two NS vs. MS clusters (reported in Table 2), were extracted for each participant (by transposing the group Talairach coordinates back to each participant's MRI image and its co-registered SAM volume). Then, these values (for each voxel) were sorted into two groups: participants who spontaneously reported little or no emotions (4 and 1 participants, respectively) during NS, and participants who did not (7 participants). There were no significant differences between the groups for both tested voxels (2-sample *t*-test, *ns*), with the PSC means of the decreased emotionality group being actually higher than the normal group (contrary to what could be expected if the tested hypothesis was correct).

#### Attentional demands

To rule out confounds resulting from attentional demands being different for the different tasks, we ran some additional analyses (on top of the subjective ratings of task success which evidenced no significant differences). The bulk of the meditation-related literature (see Cahn and Polich, 2006 for an extensive review) reports changes in anterior and posterior alpha and/or mid-line theta oscillatory activity as measures gauging the concentrative attention-related aspects of meditation. Thus, we checked (using a robust, cluster-based non-parametric permutations approach, Maris and Oostenveld, 2007) whether any significant clusters in these frequency bands could be identified. None where found. In addition, activity in the dorsolateral PFC has been specifically found to reflect task difficulty, in particular regarding long-term meditators (Brefczynski-Lewis et al., 2007). The fact that the present results showed no sign of dorsolateral PFC activity changes between the conditions can be taken as yet another indication that attentional demands do not account for the reported results.

## Discussion

In summarizing the findings of the present study, three main points emerge: (1) NS attenuation is characterized by decreases in high-gamma (60–80 Hz) oscillatory activity. These are left-hemisphere-dominated and manifest in frontal, thalamic and extensive dorsal and ventral mPFC regions, in line with the related fMRI literature; (2) MS attenuation is characterized by decreases in beta-band (13–25 Hz) oscillatory activity in both overlapping (with the gamma network) regions including the left ventral mPFC and thalamus, and a right pre-motor region, and non-overlapping regions including the right PCC and precuneus medially, and bilateral but right-hemisphere dominated IPL. While these regions have been previously tied to MS processing, the frequency band hosting these deactivations—the beta band—is a novel finding of the present study; (3) Phenomenal characterization of participants' descriptions of their SL experiences yielded three distinct categories of experience. In particular, the LO group, whose experiences indicated a sharp attenuation of the sense of agency/ownership, and who were also distinct in terms of their greater meditative expertise, also evidenced a distinct neural signature characterized by a further attenuation of the right IPL and left dorsomedial thalamus in the beta band. The implications of these results are discussed in the following paragraphs.

### NS attenuation is linked to decreased mPFC cortical activity and decreased negative emotions

As predicted, frontal, and especially medial prefrontal, high-gamma-band decreases in oscillatory activity resulted from attenuating the narrative mode of processing toward a minimal experiential mode (NS vs. MS, Figure 4). The link between NS attenuation and reduced mPFC activity, is, as noted, supported by virtually all fMRI research and review studies regarding self-referential processing (Gusnard et al., 2001; D'Argembeau et al., 2005; Northoff et al., 2006; Christoff et al., 2011; Qin and Northoff, 2011; Whitfield-Gabrieli et al., 2011; Kim, 2012). Also, as mentioned, intracranial EEG studies (Nir et al., 2007; Jerbi et al., 2010; Ossandón et al., 2011; Ramot et al., 2012) correlate self-referential and DMN blood-oxygenation-level-dependent (BOLD) reductions to suppressed high gamma-band oscillatory activity. As existing MEG studies of the self are either event-related studies (Walla et al., 2007) or connectivity studies (Lou et al., 2010b), the present study is the first to directly bridge fMRI BOLD and frequency-dependent MEG power results in the context of self-referential processing. The robust and extensive mPFC decreased gamma-power in MS relative to NS provide further evidence regarding the neural underpinning of the BOLD fMRI results, but also, importantly, anchor the results acquired through MEG to the main fMRI body of literature.

In addition to the reduced mPFC gamma oscillations, NS attenuation was also marked by a dramatic reduction of negative and mixed (both positive and negative) emotions: from 10 participants reporting such emotions in NS to only 1 in MS and SL, respectively. These are in alignment with findings directly associating increased midline activity in DMN regions to self-related emotionality (Northoff et al., 2009; Wiebking et al., 2011). As the link between increased self-focus, mPFC activity, and mood and anxiety disorders has been previously established (for reviews see Ressler and Mayberg, 2007; Lemogne et al., 2012), the present findings supports the notion that approaching self-experience through a more present-centered focus may be critical to human well-being (Davidson, 2004). A similar conclusion was reached by Killingsworth and Gilbert (2010) who showed, based on a large-scale web-based experience sampling survey, that a wandering mind (dominating over 46% of waking experience) is less happy than a mind focused on what it is doing—regardless of the valence of the activity being engaged.

### Medial and lateral parietal beta-band oscillatory activity mediate MS processing

As mentioned, the MS network evidencing beta-band power attenuation (MS vs. SL) included posterior medial and lateral parietal regions (Figure 5), which were not part of the NS network (in both beta and gamma). The IPL and right precuneus have been found to be involved in the mediation of agency (Farrer and Frith, 2002; Farrer et al., 2003; Nahab et al., 2011), in differentiating third- and first-person perspectives (Ruby and Decety, 2001; Vogeley and Fink, 2003; Vogeley et al., 2004), and in self-other discrimination (Uddin et al., 2006, 2007). The works of Olaf Blanke and colleagues (reviewed in Blanke, 2012), summarizing extensive neuroimaging and neurological data, highlight the role of right-hemisphere-dominated lateral parietal regions in mediating the most basic aspects of self-consciousness. Damasio (1999, 2010) argues that medial parietal regions are specifically involved in MS and not in NS (parallel to Damasio's core and extended selves, respectively). Laureys et al. (1999, 2004) show impaired PCC, precuneus, parieto-temporal, and prefrontal function in vegetative state patients, and suggest that it is the PCC/precuneus that distinguishes vegetative from minimally conscious patients. Using transcranial magnetic stimulation, Kwan et al. (2007) and Luber et al. (2012) established a causal role for the mPFC, but not the precuneus and right IPL in self-evaluation, while Lou et al. (2010a) showed the causal role of the IPL but not the mPFC in self-specific processes. Finally, Philippi et al. (2012) describe a patient with preserved self-awareness, recognition and agency, but an impaired autobiographical self, following extensive bilateral damage to the insula, ACC and mPFC, with medial parietal regions left intact. Together, and in line with the present findings, a strong case can be made for dissociating parietal from prefrontal regions in regard to self-reference, linking the former with MS processing.

The main finding of the present study is the beta-band network underlying MS processing, clearly dissociable in the frequency domain from the well-documented gamma-frequency network underlying NS. This finding is not surprising when considering the related literature. The basic awareness of self and others has been most often studied within the context of the sense of agency, with two main theories attempting to account for it. The first of these is the classic or extended versions of the “comparator model” or “central monitoring theory” (Frith, 1992; Synofzik et al., 2008), which posit self-awareness to hinge on to motor optimization and control networks. The second theory is of action simulation or “mirror neurons” (reviewed in Sinigaglia and Rizzolatti, 2011), which claims that attributing actions to one's self or to others hinges on our capacity to represent action as our own motor possibilities. What is shared by both accounts is that they understand minimal self-awareness to be embodied within, or mapped onto, motor systems. Numerous studies in animals and humans, including MEG, implicate large-scale beta band fronto-parietal oscillatory networks in sensorimotor decision-making, motor planning, and motor detection (reviewed in Siegel et al., 2012). While the sense of agency has not yet been empirically tied to a specific frequency band, mirror neuron effects have been shown to occur predominantly in the beta band in MEG studies (Muthukumaraswamy and Singh, 2008). Thus, as minimal self-awareness is theorized to hinge on motor-related networks which manifest predominantly in the beta band, the logical frequency band to host MS representations is, in fact, the beta band.

### MS as an early pre-reflective process inherent to NS processing

As mentioned in the introduction, some form of self-specifying minimal processing is in operation also during NS, accounting for narrative representations experienced as *our* representations. The pre-reflective nature of MS, and on the other hand, the reflective nature of NS, suggest that when self-related stimuli appear, MS processing will begin earlier than NS. While the present design does not reveal the temporal unfolding of MS vs. NS processing, there are a number of studies which may link the present findings to the time domain. The EEG-LORETA language-based (trait adjectives in reference to self, “I,” or a close friend, “he/she”) event-related study (Esslen et al., 2008) is particularly interesting, as it not only distinguishes, in line with the present and other findings (Zysset et al., 2003; Northoff et al., 2006; Schneider et al., 2008), the dorsal and ventral mPFC as differentially involved in reflective vs. pre-reflective self processing, but also determines their activation time-course. In the pre-reflective self condition, both the ventral mPFC and the insula were activated as early as 134–170 ms post stimulus, while differential dorsal mPFC activation in self vs. other -reference was only found when averaging over the whole time-course (700 ms). These temporal distinctions are upheld by a single MEG sensor-level study of self-awareness found in the literature (Walla et al., 2007). This event-related study, also language-based, examined encoding effects of the German language equivalents of “a,” “his” and “mine,” assumed to reflect different levels of self representation. The results indicated early (200–400 ms) and late (500–800 ms) time window effects. The 2D topography of the early time window reveals differential activity in posterior central electrodes (and in a few prefrontal ones), very similar to the 13–25 Hz beta-band 2D scalp map (Figure 3). Walla et al. interpret the early window effect as indicating a stage when the perceptual object, here a word, has not yet been branded as self/non-self, or in other words, a pre-MS stage. In contrast, the 2D representation of the late time window bears striking similarity to the 60–80 Hz high-gamma 2D scalp map presented in Figure 3 (frontal left activity). The similarity between the 2D cortical maps of MS and the early window, and NS and the later time window, together with the ventral vs. dorsal mPFC differential activation which holds both in terms of temporality (early vs. late time windows) and in terms of self processing mode (MS vs. NS), argue in favor of MS reflecting an early process inherent to the cognition of NS processing.

### The neurophenomenology of mindfulness-induced selflessness

Phenomenology played a double role in the present study, guiding both its design as well as data analysis. Regarding design, this study was inspired by “front-loading phenomenological insights into experimental design” (Gallagher and Sørensen, 2006). Specifically, and like other studies (Hasenkamp et al., 2012), mindfulness was employed in the spirit expressed by Varela et al. (1991) as a “… disciplined perspective on human experience that can enlarge the domain of cognitive science to include direct experience … ” (p. 33). Requesting long-term mindfulness practitioners to produce in laboratory settings the state of SL allowed a unique view of the neural correlates specific to the “minimal” aspect of momentary experience, rendering these aspects of human experience scientifically tractable (Lutz et al., 2007).

Regarding data analysis, collecting first-person descriptions of the SL experience allowed grouping the data into three distinct phenomenological categories (Table 1): AE descriptions indicated an altered spatial/sensual perspective of self experience, while LH descriptions indicated an attenuation of experience/r. LO descriptions, on the other hand, produced by participants cultured by a qualitatively greater meditation experience (Figure 6), indicated an attenuation of the agentive/ownership aspects accompanying experience. Despite the different phenomenological descriptions, all of the participants reported similar high rates of success and stability in all the tasks (including SL) relative to their past experiences (see section 3.1). This discrepancy can be interpreted as indicating a diminished MS experience for all participants, but diminished through different strategies and accompanied by distinct phenomenological experiences. In particular, the more experienced LO group is interesting as their descriptions indicate a specific subtraction of agency/ownership from momentary experience. This distinct phenomenology was then tied to a distinct neural signature: a further attenuation of beta-band power (relative to the AE and LH groups) in the left dorsomedial thalamus and right IPL (Figure 7).

Subcortical regions have only recently begun to be incorporated into theories of self-awareness (see Northoff and Panksepp, 2008; Damasio, 2010 and Christoff et al., 2011). The reported suppressed beta power in the dorsomedial thalamus support these researchers' hypotheses regarding the crucial involvement of subcortical circuits in the mediation of primal mammalian core processes tagging phenomena as self/not-self, which then feed into higher MS cortical representations. On the cortical level, the right inferior parietal sulcus has been highlighted as a region integrating multisensory bodily signals and reflecting the conscious experience of being an “I,” a spatially localized entity corresponding to first-person perspective and identity (Ionta et al., 2011; Blanke, 2012). The IPL has also been hypothesized as a key region responsible for the sense of agency and subjective sense of control (e.g., Farrer et al., 2008; Nahab et al., 2011; Haggard and Chambon, 2012). Along with these studies, the present findings support the role of this region in reflecting one of the most astonishing features of the human mind, the subjective “self as I” aspect of conscious experience, and put forth the hypothesis—to be examined by subsequent research—that it is mediated specifically within the beta band.

### Study limitations

One limitation of the study concerns its unique participants. We acknowledge a potential lack of generalizability to non-vipassana and non-meditator general populations, in particular regarding the state of SL, which is an experience cultured by meditation practice and comprehensible from a Buddhist, but perhaps not Western, point of view (but see Metzinger, 2003). Another limitation regards the small sample of participants, especially in the neurophenomenological analysis, which yielded very small groups. Thus, the results reported here warrant replication in a larger group and in other meditative traditions. In addition, the reported phenomenological analysis is rudimentary in nature. This is partly due to the experimental conditions of interviewing participants via intercom between tasks, but partly also to the exploratory nature of the advent of translating phenomenological insights of long-established contemplative traditions into current neurocognitive terms. Future studies can build on these preliminary results and develop more sophisticated phenomenological characterizations of self and selfless modes of awareness using more rigorous qualitative/phenomenological analysis methods.

## Concluding remarks

The present study highlighted the role of frequency-dependent networks, dissociable in the frequency domain but partially overlapping in brain topography, in supporting different modes of self-processing. These results emphasize the unique contribution of MEG to the neuroimaging self-awareness literature. In addition, the present study illustrated the utility of combining first-person reports, neuroimaging, and Buddhist-inspired mind training for scientifically characterizing selflessness. Indeed, a non-trivial outcome of the present study is that long-term mindfulness meditators are actually able, under experimental conditions, to successfully produce and steadily hold a selfless mode of awareness. This state of mind, which is alien to normal non-pathological conscious experience and which has not been previously scientifically documented and neurocognitively mapped, allows a unique glance at the neural underpinnings of the more subtle and basic processes of self-awareness.

### Conflict of interest statement

The authors declare that the research was conducted in the absence of any commercial or financial relationships that could be construed as a potential conflict of interest.
